# Potential of Biochar from Wood Gasification to Retain Endocrine Disrupting Chemicals

**DOI:** 10.3390/ma16020569

**Published:** 2023-01-06

**Authors:** Claudia Carnimeo, Nicola Colatorti, Valeria D’Orazio, Pasquale Trotti, Elisabetta Loffredo

**Affiliations:** Dipartimento di Scienze del Suolo, della Pianta e degli Alimenti, Università degli Studi di Bari Aldo Moro, 70126 Bari, Italy

**Keywords:** biosorbent, sorption kinetics, sorption isotherm, xenoestrogen, endocrine disrupting chemical, herbicide, desorption

## Abstract

In this study, a biochar obtained from poplar wood gasification at a temperature of 850 °C was used to adsorb the xenoestrogens 4-tert-octylphenol (OP) and bisphenol A (BPA) and the herbicide metribuzin from water. Scanning electron microscopy (SEM-EDX) and Fourier-transform infrared (FTIR) spectroscopy were employed to investigate the surface micromorphology and functional groups composition of biochar, respectively. The study of sorption kinetics showed that all compounds achieved the steady state in less than 2 h, according to a pseudo-second order model, which denoted the formation of strong bonds (chemisorption) between biochar and the compounds. Adsorption isotherms data were described by the Henry, Freundlich, Langmuir and Temkin equations. At temperatures of 10 and 30 °C, the equilibrium data of the compounds were generally better described by the Freundlich model, although, in some cases, high correlation coefficients (r ≥ 0.98) were obtained for more than one model. Freundlich constants, K_F_, for OP, BPA and metribuzin were, respectively, 218, 138 and 4 L g^−1^ at 10 °C and 295, 243 and 225 L g^−1^ at 30 °C, indicating a general increase of adsorption at higher temperature. Desorption of all compounds, especially OP and BPA, from biochar was slow and very scarce, denoting an irreversible and hysteretic process. Comparing the results of this study with those reported in the literature, we can conclude that the present biochar has a surprising ability to retain organic compounds almost permanently, thus behaving as an excellent low-cost biosorbent.

## 1. Introduction

The growing global demand for energy and the current environmental and geopolitical crisis related to the supply of fossil fuels require the exploration and optimization of clean and renewable energy production processes. Thermochemical conversion processes of waste biomass, such as gasification, pyrolysis and hydrothermal carbonization, along with biological processes, such as anaerobic digestion, are considered relatively inexpensive and environmentally friendly solutions for energy needs. In addition to gaseous and liquid fuels, these processes release large quantities of recyclable carbon-rich byproducts, which have proved to be valid soil improvers [1,2] or very efficient biosorbents for practices of environmental remediation [3,4,5]. In addition to responding to the demand for energy, the technologies used for the treatment of biowaste represent virtuous solutions to other emergencies, such as the disposal of the enormous mass of organic solid waste, the reduction of climate-altering gases emission into the atmosphere and the implementation of circular economy.

Gasification is a promising dry biomass conversion technology that produces a synthetic gaseous mixture (syngas), a bio-oil and a carbonaceous black material known as ‘biochar’. Common operating parameters of gasification are temperatures between 700 and 1000 °C, limited oxygen atmosphere and very short retention times (few hours or less) [6,7]. Suitable feedstock is forest and agricultural residues and the organic fraction of municipal solid waste. The incorporation of biochar into agricultural soil provides a very useful supplement of organic matter, which contributes to modulate the bioavailability of phytonutrients and contaminants [8]. Due to its excellent ability to adsorb both inorganic and organic pollutants, biochar is increasingly used in soil and water remediation practices [9,10]. Chemical modifications and microbial enrichment of biochar have proven effective treatments to enhance the sorption potential of this material [11,12]. The capacity of biochar to immobilize pollutants depends on its physicochemical properties, such as porosity, micromorphology, elemental composition, surface functional groups and degree of aromatization, which, in turn, depend on the operating conditions adopted for its production. The high temperatures of the gasification process favour a large specific surface area and a high number of adsorption sites for biochar, along with a high degree of aromaticity, low H/C ratio and high C/N ratio.

Repeated soil applications of agrochemicals in conventional agriculture and the increasing use of grossly decontaminated wastewater and sewage sludge for soil fertilization have caused the widespread presence of organic xenobiotics in terrestrial and aquatic environments. Environmental pollutants include agrochemicals, industrial products and byproducts, dyes, pharmaceuticals, personal care products, surfactants and so on [13,14]. Many of these pollutants are endocrine-disrupting chemicals (EDCs), as they can interfere with the endocrine system of wildlife, especially fishes, mammals and humans, causing severe dysfunctions and disturbances to the reproductive and cardiovascular systems [15,16,17]. Most of these pollutants are persistent and highly toxic, even at concentrations of a few parts per billion in water [18].

Among EDCs, there are the xenoestrogens 4-tert-octylphenol (OP) and bisphenol A [2,2-Bis(4-hydroxyphenyl) propane, BPA], which are widely employed for the industrial preparation of daily-use manufacts, such as medical devices, adhesives, paints, electrical and electronic parts, flame retardants, food and beverage packaging and so on [19]. OP is the product of biodegradation of octylphenol polyethoxylates, which are non-ionic surfactants used in the production of paints, detergents and agrochemicals [20]. OP is widely present in wastewater and, due to its recalcitrance, is persistent in ecosystems for a long time [20]. BPA is the monomer widely used for the industrial preparation of epoxy resins and polycarbonate plastics and acts as a stabilizer for polyvinyl chloride [15]. According to a recent estimate, the global annual production of BPA is around 8 million tons [21], and even more are expected to be produced in the future. Both EDCs can be widely present in ecosystems and have serious detrimental effects on animal and human health [17].

Among the crop protection products widely used in the world, there are herbicides. The repeated and incorrect use of these compounds can compromise the self-depollution capacity of soil and, consequently, these compounds can be transferred from the soil to natural waters or be absorbed by plants and accumulated in edible organs [22]. All this causes the contamination of ecosystems and the animal and human food chains. Metribuzin (4-amino-6-tert-butyl-3-(methylsulfanyl)-1,2,4-triazin-5(4H)-on) is a triazinone chemical used in huge amounts around the world to control broadleaf weeds in various crops. The high water solubility of metribuzin makes it one of the pesticides with the highest risk of transport to ground- and surface water [23]. Metribuzin is also a suspected EDC [16].

Adsorption consists of the accumulation of a solute at the interface between the adsorbent phase and the solution phase. In soil, the adsorption/desorption process controls the fate of contaminants, including movement and persistence, and modulates their bioavailability for plants and microorganisms. The incorporation of organic materials, like biochar, into the soil can hinder the transport of contaminants into natural water bodies. This is particularly important in the case of recalcitrant molecules [24]. Furthermore, the need to decontaminate wastewater for virtuous recycling nowadays requires the abandonment of complex strategies and expensive materials. In recent years, biochar has shown excellent adsorption capacity of both inorganic and organic pollutants [5,25]. Among the studies available in the literature on the sorption/desorption of organic pollutants by biochar, a very limited number concern biochar produced by gasification. This process is generally conducted at much higher temperatures than those adopted in the more common pyrolysis, and this can greatly influence both the properties of biochar and its efficiency in retaining pollutants. Furthermore, information on the ability of biochar to remove phenolic xenoestrogens, such as BPA and OP, is very scarce. Finally, few studies have focused on the removal of ECDs from multi-contaminated matrices [13], although multi-contamination is very frequent in water and soil where pollutants with different hydrophobicity coexist and interact with solid and dissolved soil components.

The present study aims to evaluate the capacity of a biochar from wood gasification to adsorb three EDCs with contrasting physicochemical properties, namely OP, BPA and metribuzin.

## 2. Materials and Methods

### 2.1. Chemicals and Biochar

**Figure 1:**
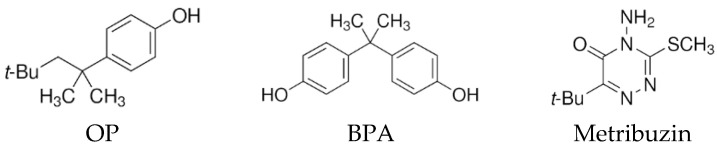
Chemical structures of the compounds.

The compounds OP, BPA and metribuzin have, respectively, a molecular weight of 206.32, 228.29 and 214.29 g mol^−1^, a water solubility of 3.1, 300 and 1200 mg L^−1^ and a Log Kow of 5.50, 3.32 and 1.70 [26]. The chemical structures of the compounds are shown in Figure 1. OP at 99.5% purity, BPA at 99.0% purity and metribuzin at a purity ≥98.0% were obtained from Sigma-Aldrich S.r.l., Milan, Italy. All other chemicals used were of extra pure grade and obtained from commercial companies. Methanol solutions of OP, BPA and metribuzin were prepared at a concentration of 2000 mg L^−1^. Then, appropriate volumes of each solution were combined and diluted with double distilled water to obtain the aqueous mixtures of the compounds used in the experiments. The most concentrated mixture of the compounds used in this work (2 mg L^−1^) had a methanol content of 0.3%.

The biochar sample was produced through a high-temperature (850 °C) gasification process and the SyngaSmart^®^ technology of the RESET s.p.a. company, Rome, Italy. Chipped and dried wood from the poplar clone Monviso was used as feedstock.

### 2.2. Biochar Characterization

#### 2.2.1. Basic Characterization and Elemental Analysis 

Basic characterization of biochar was carried out according to conventional methods and is shown in Table 1. Elemental composition was obtained using a CHNS-O Elemental Analyser, as described in Taskin et al. [27]. 

#### 2.2.2. Scanning Electron Microscopy (SEM) Analysis

To investigate biochar micromorphology, SEM analysis coupled with energy-dispersive X-ray spectroscopy (SEM-EDX) was performed. For the purpose, a little amount of biochar was metallized with Au/Pd and analyzed using a Hitachi TM3000 scanning electron microscope (Hitachi, Tokyo, Japan) and an Oxford Swift ED3000 microanalysis system. Backscattered electrons were detected, and SEM micrographs of biochar were obtained at both 500× and 1800× magnifications.

#### 2.2.3. Fourier Transform Infrared (FTIR) Analysis

To evaluate surface functional groups of biochar, the FTIR spectrum was acquired in transmittance mode. A mixture of 400 µg of biochar and 400 mg of KBr (FTIR grade) was finely ground in an agate mortar. The mixture was then pressed under vacuum at a pressure of 6000 kg cm^−1^ for 10 min, thus obtaining a thin pellet. The sample was analyzed using a Thermo Nicolet iS50 FTIR spectrophotometer equipped with Nicolet Omnic 6.0 software. Spectrum acquisition conditions were: wavenumber between 4000 and 400 cm^−1^, 2 cm^−1^ resolution and 64 scans min^−1^.

### 2.3. Preliminary Adsorption Experiments

Aliquots of 1, 2, 5, 10 and 20 mg of biochar were interacted with a volume of 10 mL of an aqueous mixture of OP, BPA and metribuzin, each at a dose of 2 mg L^−1^, which corresponded to solution/adsorbent ratios of 10,000, 5000, 2000, 1000 and 500, respectively. To achieve the adsorption equilibrium, the samples were placed under magnetic stirring at 310× *g* for 120 min at room temperature (26 ± 1 °C). The experimental time of 2 h for equilibrium was established in previous trials. After that, the samples were centrifuged (10,000× *g* for 10 min) and supernatants were analyzed by ultra-high performance liquid chromatography (UHPLC) (see Section 2.6). Three parallel experiments were carried out to obtain the average and error estimates. At the end of experiments, the concentration of the adsorbed molecules, q_t_ (mg g^−1^) were calculated using the equation: q_t_ = (C_0_ − C_t_) × V/m, where C_0_ (mg L^−1^) is the starting dose of the molecule in solution, C_t_ (mg L^−1^) is the dose at time t (120 min in these experiments), V (L) is the solution volume and m (g) is the adsorbent mass.

### 2.4. Adsorption Kinetics

To determine the adsorption rates of OP, BPA and metribuzin onto biochar and to establish the equilibrium time, sorption kinetics were performed at room temperature (26 ± 1 °C). In these experiments, the solution/adsorbent ratio of 10,000 was adopted. Volumes of 20 mL of an aqueous mixture of the three molecules, each at dose of 2 mg L^−1^, were interacted with 2 mg of biochar in glass centrifuge tubes. The suspensions were stirred in the dark for time periods ranging from 0 to 120 min. Subsequently, the samples were processed and analyzed as described in Section 2.3. Each experiment was conducted in triplicate. Using the equation reported in Section 2.3, the concentration of the adsorbed compound after a time t, q_t_ (mg g^−1^), was calculated. The equilibrium time was established when at two successive times the quantity of compound adsorbed was unchanged according to the Student’s *t* test (*p* ≤ 0.05).

Sorption kinetics data were fitted into the pseudo-first order (PFO) [28,29] and the pseudo-second order (PSO) [30] equations, which allowed calculation of the kinetic constants and obtaining indications of the type of molecular interaction between the biochar and the compounds. Table 2 shows the two theoretical models, along with the corresponding parameters that were calculated with the non-linear regression method. The accordance between the experimental data and each model was estimated by the correlation coefficient: $r=\sqrt{\frac{\sum {\left(q_{t}m-\bar{q_{t}}\right)}^{2}}{\sum {\left(q_{t}m-\bar{q_{t}}\right)}^{2}+\sum {\left(q_{t}m-q_{t}\right)}^{2}}}$, where q_t_m is the theoretical concentration of the adsorbed compound (mg g^−1^) at time t, q_t_ is the experimental concentration (mg g^−1^) and $\bar{{\text{q}}_{\text{t}}}$ is the average q_t_.

### 2.5. Adsorption Isotherms

Adsorption isotherms of the compounds onto biochar were conducted at two different temperatures, 10 and 30 °C, using the slurry-type mode. Volumes of 20 mL of aqueous mixtures of OP, BPA and metribuzin, each at doses of 0.1, 0.2, 0.4, 0.5, 1 and 2 mg L^−1^, were added to aliquots of 2 mg of biochar in glass centrifuge tubes. Samples were kept in a thermostated chamber (F.lli Della Marca S.r.l., Rome, Italy) under magnetic stirring in the dark for 120 min and subsequently processed and analyzed, as reported in Section 2.3. All experiments were performed in triplicate.

Desorption experiments were conducted using 2 mg of biochar interacted with a volume of 20 mL of an aqueous mixture of OP, BPA and metribuzin at individual concentration of 2 mg L^−1^. Desorption started soon after adsorption and was carried out for four desorption steps. At each desorption cycle, a volume of 16 mL of equilibrium supernatant solution was replaced with the same volume of distilled water. After stirring the sample for an additional 24 h at room temperature (25 ± 1 °C) and processing it in the conditions described in Section 2.3, the residual concentration of the compounds was analyzed in the supernatant solution by UHPLC (see Section 2.6).

Different models were used to interpret adsorption isotherms data, namely the nonlinear Freundlich, Langmuir and Temkin models and the linear Henry model. The equations and corresponding parameters are shown in Table 2. The Freundlich parameters, K_F_ and 1/n; the Langmuir parameters, b and K_L_; and the Temkin parameters, B and A_T_, were all calculated by the non-linear regression method, which allowed minimizing the sum of squared residuals (SSR) between experimental and theoretical data. The accordance between the experimental data and each model was estimated by the r value, as described in Section 2.4. Finally, the linear Henry model assumes that, during the adsorption process, there is a constant distribution of the solute molecules between the solution and the substrate over the concentration range tested. The Henry equation allows calculating the distribution coefficient, K_d_, from the slope. The organic-carbon-partition coefficient, K_OC_, which expresses the amount of compound adsorbed per unit of organic carbon (OC) of the substrate, was calculated by: K_OC_ = (K_d_ × 100)/(% OC)) [31].

### 2.6. Analytical Measurement

Before UHPLC analysis, each sample was filtered through 0.45 μm Millipore^TM^ cellulose acetate filters. A Dionex Ultimate 3000 RSLC (Waltham, MA, USA), equipped with an HPG-3200 RS pump, a WPS-3000 autosampler and a TCC-3000 column compartment connected to a Supelco^TM^ LC-18 column (250 mm × 4.6 mm × 5 μm) was used. Water (A) and acetonitrile (B) were used to prepare the mobile phase, which flowed at 0.8 mL min^−1^. The programmed gradient elution was: 0–7 min, 60% B; 7–15 min, from 60 to 90% B. Retention times of OP, BPA and metribuzin were, in order, 13.9, 5.8 and 4.2 min. A FLD-3400 RS fluorescence detector (Dionex Ultimate 3000 RSLC, Waltham, MA, USA) operating at wavelengths of 230-nm excitation and 310-nm emission was used to detect the two phenols, while a DAD-3000 RS diode array detector (Dionex Ultimate 3000 RSLC, Waltham, MA, USA) at a wavelength of 294 nm was used to detect metribuzin.

## 3. Results and Discussion

### 3.1. Biochar Characterization

#### 3.1.1. Basic Characterization and Elemental Analysis

Basic properties of the biochar sample (Table 1) are comparable to those reported in the scientific literature for wood biochars from pyrolysis or gasification [5]. As expected, biochar showed high values of pH, EC and ash content. Gasification causes a rearrangement of the functional groups of the raw material due to the dehydration, decarboxylation and aromatization processes that occur with the rise in temperature, and that generally leads to an increase of pH [32].

The elemental analysis provided the elemental composition of biochar and allowed calculating the atomic ratios of elements (Table 1). It is known that both the feedstock and the operating conditions adopted in biochar production play a relevant role in its basic properties and elemental composition. The C, N, H and O contents were comparable with those reported for other wood biochars obtained by gasification [33]. The high C content of the material is advantageous for both C storage and adsorption of contaminants. During the thermochemical conversion of biomass, intense dehydration and decarboxylation processes cause a marked increase of C content and a decrease of O and H content, compared to the original biomass [32]. The atomic H/C, O/C and (O + N)/C ratios are important parameters for evaluating, respectively, the degree of carbonization, hydrophilicity and polarity index of the material, which strongly depends on the process temperature. The low H/C ratio of this biochar (0.14) suggests a highly condensed aromatic structure and a marked thermal degradation [9,30]. Furthermore, the low H/C ratio, along with the high C/N ratio (166.90), of this biochar is indicative of intense carbonization with abundant loss of N and H, compared to C [30].

#### 3.1.2. SEM Analysis

**Figure 2:**
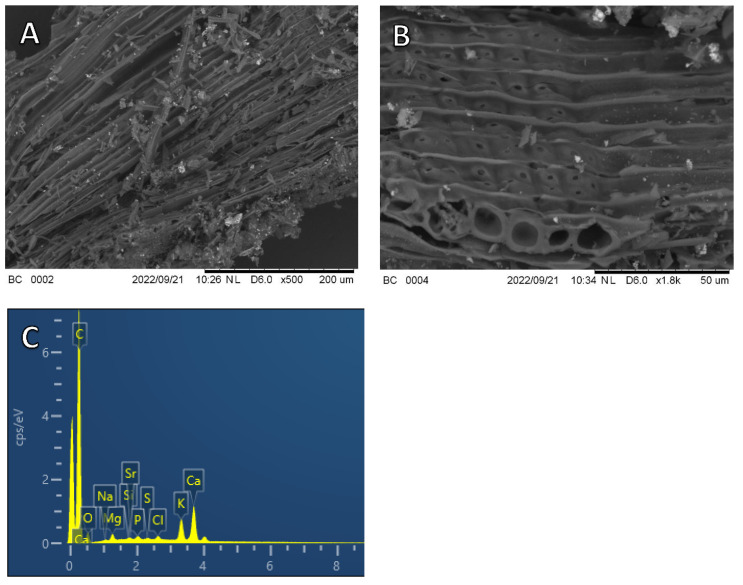
Scanning electron microscopy (SEM) images at magnifications of 500 (**A**) and 1800 (**B**) and energy-dispersive X-ray (EDX) spectrum (**C**) of biochar. Images were taken with secondary electrons.

The micromorphological aspects of the biochar surface and information on the distribution and allocation of the pores in the material were investigated using the SEM technique coupled with EDX elemental analysis. Images were obtained at 500× and 1800× magnifications (Figure 2). SEM images of the biochar clearly revealed a rough surface with nearly regular ridges, channels and cavities originating from the cell walls and vascular tissues of poplar wood used as feedstock for the gasification process (Figure 2A,B). The presence of numerous pores is due to the volatilization of material during gasification. Microparticles, mostly of few µm, and small pores of different diameter, nearly or less than 10 µm, were also present (Figure 2A,B). Porosity and a large surface area of the adsorbent are extremely important properties for the adsorption of organic molecules. Biochar porosity originates from the loss of small volatile molecules, such as H_2_O, CO, CO_2_ and CH_4_, during the thermochemical conversion of biomass [27]. In a previous work of Taskin et al. [27], SEM images of two wood biochars showed a wide porosity, but not the original vascular structures of the plant. This might be due to the lower temperature and the longer residence time of the pyrolysis process that originated those biochars, compared to this biochar, which drastically altered the structure of the starting woody biomass. 

The EDX spectrum evidenced the presence on the biochar surface of various elements, such as Ca, Mg, Na, K, P and so on, that are typical of plant-based materials (Figure 2C). During the gasification process, the alkali metals K and Na are retained in biochar, which explains the high pH and EC values observed. Ca, K, Mg and P are the most abundant elements in biochar [27]. High contents of these elements were found in biochar from red spruce pellets [27] and chopped red cedar wood [34].

#### 3.1.3. FTIR Analysis

**Figure 3:**
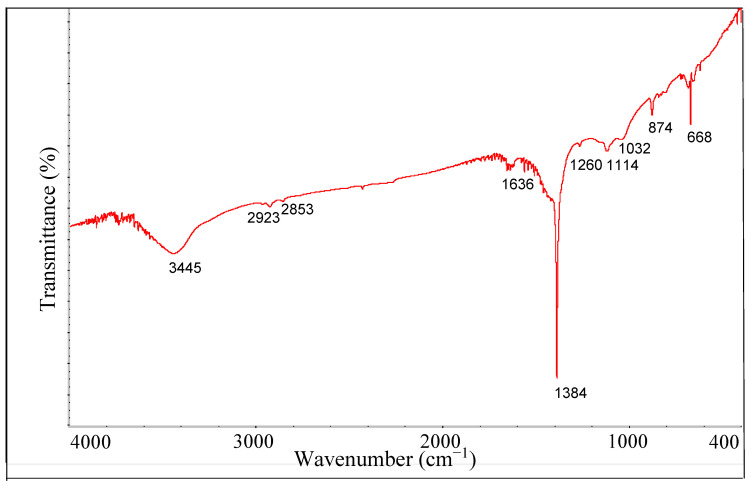
FTIR spectrum of biochar.

To investigate the chemical structure and surface functional groups of biochar, FTIR analysis was performed. Overall, the FTIR spectrum (Figure 3) of the biochar was characterized by a poor chemical diversity with few absorption bands, generally of very low intensities, which denotes an aromatic nature, but with low H content (see Table 1). The shift of the baseline on the y axis suggests that dehydrogenation mechanisms and subsequent rearrangement and polymerization of carbonaceous aromatic units have occurred during biochar formation. In detail, the absorption frequencies (cm^−1^) of the biochar spectrum and the relative assignments are as follows: 3445 cm^−1^, O-H stretching of hydroxyl groups and C-H stretching of 5-membered N/O-heterocyclic C (e.g., furans and pyrroles); 2923–2853 cm^−1^, C-H asym and sym stretching of CH_2_ groups; 1636 cm^−1^: C=C aromatic skeletal vibration; 1384 cm^−1^, in-plane bending of phenolic –OH, likely related to ligneous syringyl units, and furanosic-like structures; 1260 cm^−1^, C-O stretching of phenolic groups, indicative of guaiacyl units associated with lignin; 1114 cm^−1^: sym C-O-C stretching vibrations in cellulose and hemicellulose and/or aliphatic -OH; 1032 cm^−1^, sym stretching of acid derivatives, aliphatic C-O-C and –OH representative of oxygenated functional groups of cellulose and hemicellulose and methoxy groups of lignins; 874 and 668 cm^−1^, aromatic C-H out-of-plane bending [35,36]. It is evident that, due to the extended carbonization at 800 °C, the aromatic moieties result as prevalent, likely indicating the formation of graphite-like structures, with low amounts of oxygenated/hydrogenated groups. These results agree with data of elemental analysis that indicate low H/C, O/C and (O + N)/C atomic ratios, corresponding to the absence or limited presence of oxygenated functional groups on the surface and high percentages of carbon content. The FTIR spectrum obtained was similar to that of a char obtained from red cedar through gasification at 800 °C [34]. 

### 3.2. Preliminary Adsorption Experiments

**Figure 4:**
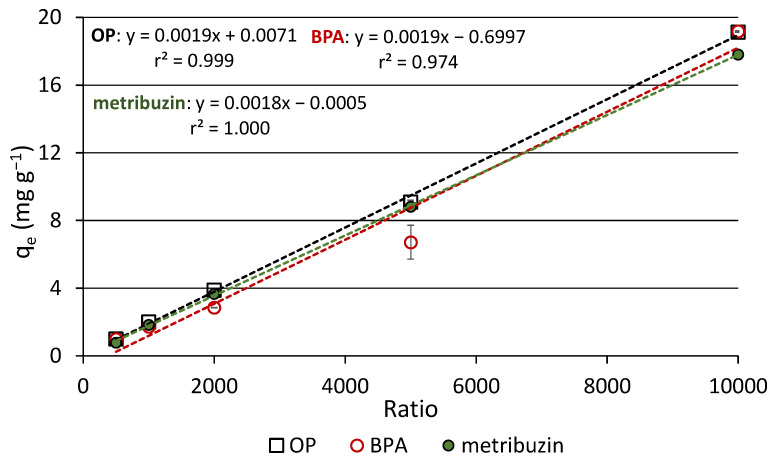
Effects of the solution/biochar ratio on the adsorption of the compounds at the equilibrium condition.

The sorption efficiency of biochar was investigated at five different solution/adsorbent ratios. The amounts of the compounds adsorbed on the substrate unit, for each ratio, after an equilibration time of 120 min are reported in Table 3 and Figure 4. The different solution/adsorbent ratios used are quite common in adsorption studies using biochar [37,38]. The determination coefficients, r^2^, obtained by the linear regression of equilibrium data of each compound were very high, indicating the occurrence of a linear relationship between the concentration of the adsorbed compound and the ratio adopted (Figure 4). Statistical analysis of the concentrations of adsorbed compound at the different ratios evidenced for all compounds highly significant increases (*p* ≤ 0.01) at each subsequent higher ratio tested, with the only exception for BPA at the ratios of 500 and 1000 (Table 3). The same trend was observed for the three compounds, despite their different hydrophobicity. At the highest ratio (10,000), the percentages of OP, BPA and metribuzin adsorbed on biochar at equilibrium were, respectively, 95.62, 95.88 and 89.03% of the initial compound added (20 μg). Based on these results, the highest ratio was chosen for the subsequent sorption experiments.

It can be hypothesized that at higher solution/adsorbent ratios, more sorption sites of biochar were accessible/available for the solute, including the innermost ones, compared to lower ratios. In a very recent work, Islam et al. [39] investigated the effects of biochar dosage on the adsorption capacity of methyl orange and found that by increasing the dosage of the adsorbent, for the same volume of solution, the removal of the dye from water significantly decreased, thus suggesting an optimal dosage of 0.5 g L^−1^, which corresponded to a solution/biochar ratio of 2000. Considering the physicochemical properties of the compounds, as expected, the affinity for biochar was higher for the two phenols than for the less hydrophobic and much more water-soluble metribuzin. A negative correlation was previously demonstrated between the sorption efficiency of biochar and the water solubility of some EDCs and pesticides [40]. 

### 3.3. Adsorption Kinetics

**Figure 5:**
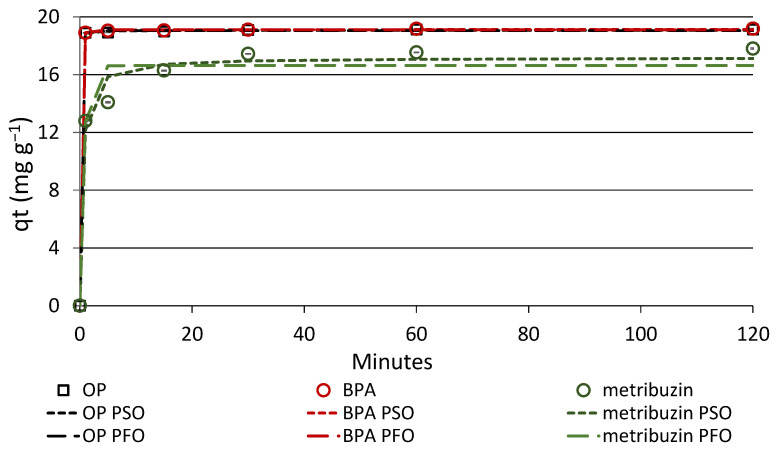
Adsorption kinetics data and plots of predicted PFO and PSO kinetics of the compounds onto biochar. Standard error is reported as vertical bar on each point (n = 3).

Adsorption kinetics were performed to estimate the retention rate of the three compounds onto biochar and to investigate the prevalent type of interaction. Adsorption kinetics data are shown in Table 4 and Figure 5. All compounds reached the steady state in a very short time, i.e., a few minutes for OP and BPA and about 30 min for metribuzin (Figure 5). At equilibrium time, the concentrations of adsorbed OP and BPA were almost identical and equal to about 19 mg g^−1^, whereas the metribuzin concentration was about 17 mg g^−1^ (Figure 5 and Table 3). The longer equilibrium time and lower concentration of adsorbed metribuzin, compared to the phenolic EDCs, indicated a lower affinity of this molecule for biochar, which probably depends on its lower hydrophobicity. Differently from that observed for other wood biochars [38], the adsorption of metribuzin by this biochar was relevant and quantitatively similar to that of OP and BPA. This discrepancy most likely depends on the production temperature of the material, which was much lower in that study (550 °C). An equilibrium time of 120 min was adopted in the adsorption isotherms experiments.

Based on the shape of the kinetic curve, we can assume that the adsorption of OP and BPA was almost instantaneous, while that of metribuzin was a multi-step process consisting in an initial rapid adsorption on the most accessible external sites of biochar, followed by a slower adsorption on the innermost active sites. The greatest removals were observed for the more hydrophobic OP and BPA (Table 4 and Figure 5). 

Information on the adsorption mechanisms of the compounds onto biochar were obtained by fitting kinetic data into the non-linear PFO and PSO equations. Both models are commonly adopted in this type of study [29,30]. The PFO model of Lagergren [28] highlights the relevant role of the adsorbent surface, as it theorizes the occurrence of a linear relationship between the number of sites available on the adsorbent and the speed of their occupation by the solute [29]. The PFO equation is well suited to describe a physisorption process. Differently, the PSO kinetic model considers in particular the type of adsorption at equilibrium and theorizes the formation of chemical bonds between the solute and the adsorbent [30]. Then, the PSO model is appropriate when the solute binds to the adsorbent through covalent bonds (chemisorption), which is the rate-limiting step of the process.

Table 4 shows the values of the kinetics parameters obtained for the three compounds according to the PFO and PSO models, along with the correlation coefficients, r, and the sum of squared residuals, SSR. Based on the values of r and SSR, the PSO model was the preferential fit for all compounds (Table 4). The experimental kinetics data and plots of the predicted PFO and PSO kinetics are shown in Figure 5. These results suggest that strong covalent bonds were formed during the sorption process, possibly coexisting with weak bonds, such as van der Waal forces and hydrogen bonding that are typical of physisorption. In a previous study, OP adsorption onto a red spruce biochar followed the PSO equation very well [40]. Kinetic sorption data of BPA on a grapefruit peel biochar fitted the PSO model well, being the adsorption mechanism controlled by forces such as π-π electron donor-acceptor bond, H-bond and others, of which chemisorption was the rate control step [41]. A prevalent π-π electron donor-acceptor binding between BPA and biochar was found by Xu et al. [42]. Hydrogen bonds and Coulombic forces were reported by Essandoh et al. [43] as the main mechanisms of metribuzin adsorption on biochar, along with weaker bonds, such as van der Waal and dipole-dipole interactions. The adsorption of metribuzin onto two biochars produced from wood residues was better interpreted by the PSO equation [38].

### 3.4. Adsorption Isotherms

Adsorption isotherm study allows estimating the adsorption parameters and provides information on the type of allocation of a compound onto biochar. To investigate the effects of temperature on adsorption, slurry-type experiments were conducted at temperatures of 10 and 30 °C. Isotherm data obtained for each compound and each temperature were interpreted with the equations of Henry, Freundlich, Langmuir and Temkin. Modeling of isotherm data provides indication of the adsorption mode of the solute on the substrate. The non-linear Freundlich model is appropriate for solutes that form multilayer adsorption on a heterogeneous substrate and does not assume substrate saturation. Conversely, the Langmuir model is proper for homogeneous materials and when there is negligible molecular interaction between the adsorbed molecules that form a monolayer on the adsorbent. The Temkin isotherm predicts a logarithmic reduction of available sites and sorptive energy involved and is best applied at intermediate concentrations of the solute. 

**Figure 6:**
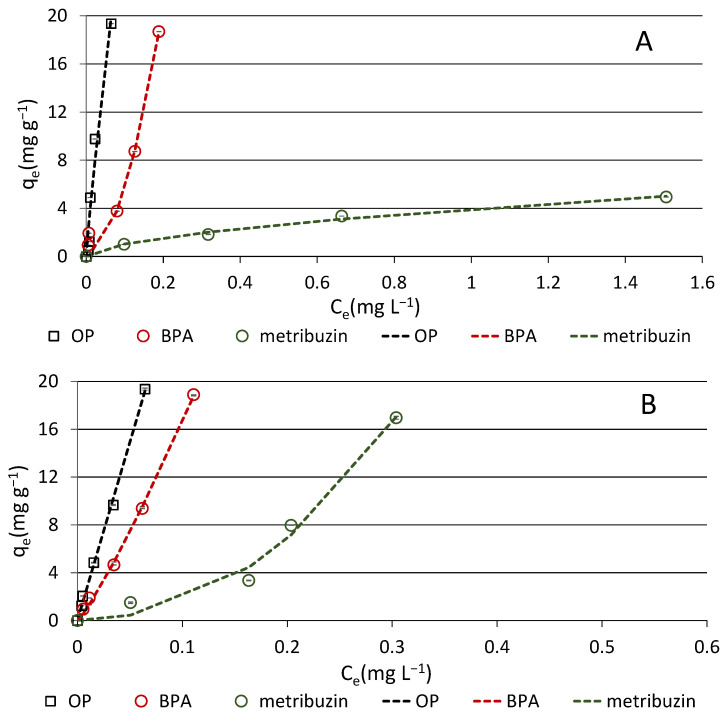
Adsorption isotherms of the compounds onto biochar conducted at temperatures of 10 °C (**A**) and 30 °C (**B**). Experimental points are shown along with plot (dashed lines) of the Freundlich model. Standard error is reported as vertical bar on each point (n = 3).

The isotherm parameters obtained by fitting the equilibrium data in all models are given in Table 5, while the experimental data, along with the plots of the predicted Freundlich model, are depicted in Figure 6. Based on both r and SSR values, in general, at both temperatures, the best fit for the compounds was the Freundlich model (Table 5). In particular, at the temperature of 10 °C, all three compounds followed the Freundlich equation (lowest SSR values) very well, although OP data were also well described by the Henry and the Temkin equations (Table 5). This finding is confirmed by the values of the Freundlich exponent (1/n), which indicate that, according to Giles et al. [44], the isotherm of OP was nearly C-type (1/n~1), that of BPA was S-shaped (1/n > 1) and that of metribuzin was L-shaped (1/n < 1) (Table 5). A linear C-type isotherm assumes a constant partitioning of the solute between the solution and the adsorbent, without reaching saturation in the concentration range adopted. A S-type isotherm indicates an increase in the rate of adsorption, with increasing solute concentration in the aqueous medium. Finally, an L-type isotherm describes the adsorption of a compound having high affinity for the adsorbent at low solute concentration, and in the initial stage of adsorption, while successively, as the surface sites are occupied, the rate of the process decreases without reaching the saturation of the adsorbent. The L-shaped isotherm is typical of low hydrophobicity solutes, such as metribuzin (log Kow = 1.70), on heterogeneous substrates, such as biochar. The adsorption of metribuzin onto a wood biochar was well interpreted by L-shaped Freundlich isotherms [34]. The Freundlich exponent 1/n is a non-linearity index and expresses the strength of adsorption, while its reciprocal n is the heterogeneity factor. The 1/n values can also give information on the mechanism of adsorption. When 1/n < 1, the solute is mainly adsorbed by physical interaction, while when 1/n > 1, the chemical bond prevails [40]. Thus, we can assume that an important role was played by chemical bonding in BPA (1/n > 1) adsorption, physical interaction in metribuzin (1/n < 1) adsorption and both types of bonding in OP (1/n~1) adsorption.

When the sorption isotherms were performed at a temperature of 30 °C, once again, all compounds showed high r values and low SSR values for the Freundlich model. Hence, the adsorption of each compound occurred through the formation of a multilayer of molecules on the heterogeneous surface of biochar. At this temperature, both for OP and for BPA, high correlation coefficients were obtained also for the Henry equation, which agrees with their 1/n values equal or close to the unit (C-type isotherm).

Therefore, it is plausible that a constant partitioning of OP and BPA occurred between the adsorbent and the solution as the solution concentration increased (Table 4). The highest discrepancy between theoretical and experimental data was observed for BPA and the Temkin equation at both temperatures tested.

**Figure 7:**
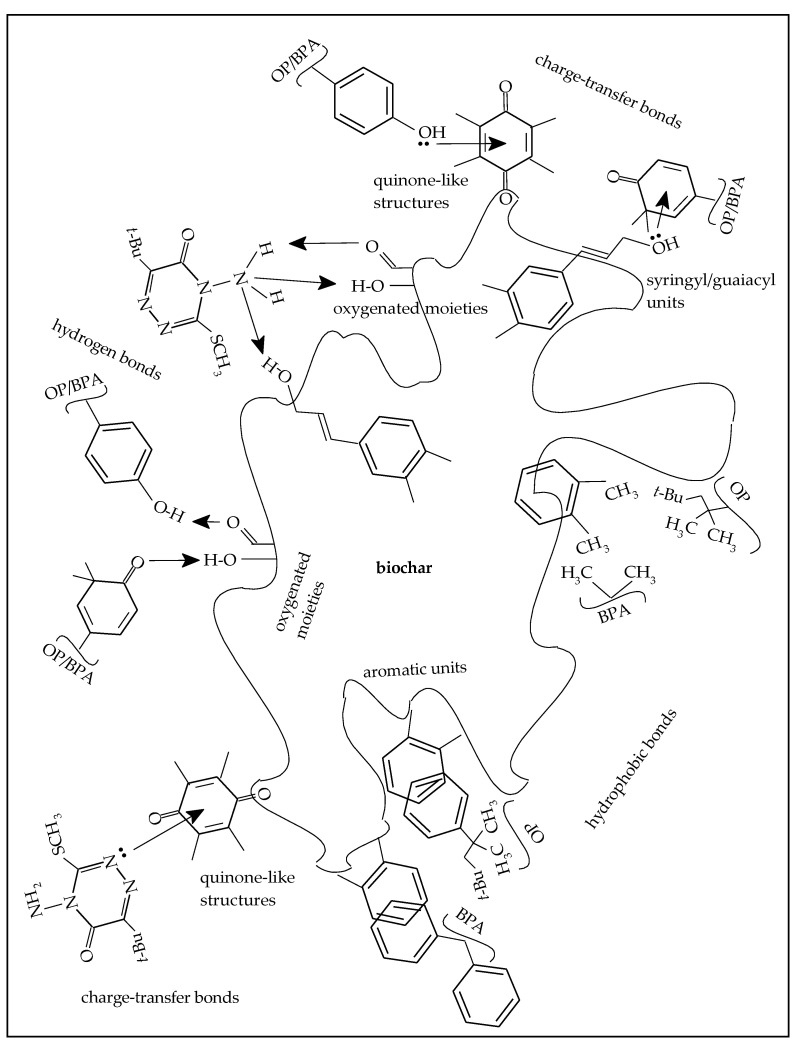
Adsorption mechanisms of the compounds onto biochar.

The adsorption of organic compounds onto C-rich material, such as biochar, occurs through physical (physisorption) and chemical (chemisorption) interaction mechanisms and forces of various strength. Physisorption is a low-enthalpy and reversible process that occurs when the solute binds to the adsorbent through weak interactions, such as van der Waals forces and H bonding. Differently, chemisorption is a high-enthalpy and almost irreversible process, as it includes strong interaction between the solute and the adsorbent through valence forces, such as covalent or ionic bonds. Adsorption of EDCs onto C-rich materials is most likely to occur through various mechanisms that depend on the extent and type of functionalities of the adsorbent, which, in turn, are dictated by the operating parameters adopted in the production process [45]. Low process temperatures allow the formation of O-containing groups on biochar, while high temperatures favor carbonization and aromatization reactions with a consequent high degree of hydrophobicity and prevalent hydrophobic interaction with organic compounds [45]. The high temperature of the gasification process originating the biochar sample of this study favoured the formation of a very great number of sorption sites on the material, which allowed high efficiency of retention of a wide range of molecules with contrasting properties, such as the ones tested in this study. Therefore, the three EDCs can be adsorbed on both hydrophilic and hydrophobic active sites, depending on their specific chemico-structural characteristics (Figure 7). As is well known, the pH value chosen in the experimental conditions strongly influences the adsorption mechanisms, as it induces important modifications of the physicochemical properties of the molecules, and, consequently, of their ability to interact with the adsorbent surface. In our study, the pH value of the suspension biochar-solution was equal to 8.3 and, consequently, BPA and OP, which are weak organic acids (pKa, respectively, 9.78/10.39 and about 10), were mostly undissociated. 

Likely, charge-transfer bonds (π-π) via electron donor-acceptor mechanisms are formed between activated electron-donor molecules, such as the phenolic units of BPA and OP, and deactivated electron-acceptor moieties occurring on the biochar surface, such as quinone-like structures (Figure 7) [46,47]. Furthermore, as in phenols the OH group is linked to an sp2 hybridized carbon, an equilibrium is created between the alcoholic form and the respective carbonyl form, i.e., in aqueous solution, both the alcoholic form and the quinoid form are present. In this case, the charge-transfer bond would take place between electron-acceptor-deactivated quinoide rings of BPA/OP and activated electron-donor molecules, such as syringyl and guaiacyl units occurring on the biochar surface. Hydrogen bonds are likely involved in the adsorption of BPA and OP, as both molecules are characterized by the presence of hydrogen donors and acceptors (Figure 7) [47]. Furthermore, considering the hydrophobic skeleton of BPA and OP molecules and the carbonaceous aromatic units present on biochar surface, it is reasonable that an adsorption on the biochar occurred through non-specific hydrophobic bonds (Figure 7) [48]. In general, the higher degree of adsorption of OP compared to that of BPA, as evidenced by equilibrium data, is probably due to a higher contribution of non-specific hydrophobic bonds due to the larger alkyl side chain. Likely, the molecules tend to enter the biochar as a result of their small sizes (e.g., molecular size of BPA 4.36 Å), and the match between the pore size of biochar and the molecular size of the adsorbates plays a key role in the adsorption [45,49]. Covalent and H bonds were reported as the prevalent bonds occurring in the adsorption of OP onto a wood biochar [40]. 

The adsorption of metribuzin onto a switchgrass biochar was mainly ascribed to H bonds and Coulombic forces and, to a lesser extent, to van der Waal and dipole-dipole interactions [43]. The presence of O-containing sites on the surface of biochar, although limited in number due to the conditions of biochar production, allowed the formation of H bonds with metribuzin, mainly involving the guaiacyl and syringyl units of the biochar and the amino group present on the triazine ring (Figure 7). Further adsorption mechanisms may be hydrophobic bonding between the alkyl side chains of metribuzin and the hydrophobic sites on biochar surface. Finally, it should be taken into consideration the formation of charge-transfer bonds between the electron-donor ring of metribuzin and electron-accepting units present on the biochar surface, e.g., quinones (Figure 7).

In this study, a remarkable adsorption of all compounds, especially the more hydrophobic OP and BPA, was shown by biochar. Using the Henry equation, it was possible to calculate the distribution coefficient, K_d_, that expresses the sorption efficiency of a substrate, and the organic-carbon-partition coefficient, K_OC_, that is a measure of the amount of compound adsorbed per unit of organic carbon of the substrate. Based on the K_F_ values obtained in the experiments conducted at 10 °C, the adsorption capacity of biochar for the three molecules followed the order: OP > BPA > metribuzin (Table 5). The same order was observed for K_d_ and K_OC_ values. As expected, the most hydrophobic OP and BPA were the most adsorbed by biochar, which confirms the general high affinity of this material for low polar molecules. At this temperature, the K_F_ and K_OC_ values of OP were two orders of magnitude higher than those of the more water-soluble metribuzin (Table 5). The maximum adsorptions, expressed by the parameter b of the Langmuir equation, followed the same order as the Henry and Freundlich constants (Table 5). The estimated parameters of the Temkin equation are A_T_, B and b_T_ (Table 5). Considering the r values, only the experimental data of OP matched the Temkin equation (r = 0.988) quite well, while those of the other compounds differed noticeably from this model. Parameter B gives an indication of the heat of adsorption. For each molecule, especially the most hydrophobic OP, the B value was relatively high and always higher than the unit suggesting exothermic adsorption of the molecules on biochar [50].

When the experiments were conducted at a temperature of 30 °C, the adsorption constants K_d_, K_F_ and K_OC_ followed the same order, OP > BPA > metribuzin, already observed at the lower temperature, although at 30 °C, the K_F_ values were only slightly different from each other, indicating a similar behavior of the three compounds (Table 5). The Langmuir b values (maximum adsorption) showed the trend BPA > OP > metribuzin, but the r values for this equation were not sufficiently high for all compounds. The Temkin B values were all greater than the unit, indicating, also at 30 °C, the occurrence of an exothermic interaction between biochar and the compounds (Table 5).

The values of the adsorption parameters obtained in this study at both temperatures tested were generally comparable to or higher than those reported in the scientific literature for biochar produced by pyrolysis or gasification at temperatures between 750 and 850 °C. As the production temperature noticeably influences the physicochemical properties and sorption efficiency of biochar, a comparison between this biochar and other biochars obtained at very different temperatures does not seem appropriate. The k_F_ value reported by Del Bubba et al. [51] for OP adsorption on a sawdust biochar produced at 850 °C (0.63 L g^−1^) is much lower than that observed in our study (about 218 and 295 L g^−1^ at, respectively, 10 and 30 °C). The k_F_ values observed in this work for BPA (about 138 and 243 L g^−1^ at, respectively, 10 and 30 °C) were higher than those found for BPA on biochars produced at 800 °C from pine chips (9.2 L g^−1^) [46] and from sawdust (6.5 L g^−1^) [42]. On the contrary, a k_F_ value as high as 1,408 L g^−1^ was reported for BPA adsorption on Argan nut shell biochar [52]. The relevant metribuzin adsorption demonstrated in this study is in agreement with what observed by Essandoh et al. [43] using a plant-derived biochar. Studying metribuzin adsorption onto sugarcane bagasse biochar produced at 700 °C, White et al. [53] obtained K_F_ (47.2 L kg^−1^) and K_d_ values (15.9 L kg^−1^) that were intermediate between those found here at the two temperatures tested. The 1/n value obtained at 10 °C for metribuzin was similar to that found by White et al. [53] using a sugarcane biochar (0.61) and by Loffredo et al. [38] using two wood biochars (0.55 and 0.65), which suggests similar surface heterogeneity of the materials.

At the two selected temperatures, the biochar showed the significantly different sorption capacities of the compounds (Table 5). In general, the values of the sorption constants were higher at 30 °C than at 10 °C, and this was particularly evident for metribuzin (Table 5). Compared to the K_F_ values of OP, BPA and metribuzin obtained at 10 °C, those obtained at 30 °C were, respectively, 1.3, 1.7 and 57.8 times higher. A positive influence of temperature on metribuzin adsorption on biochar was observed by Essandoh et al. [43] between 25 and 35 °C, but not between 35 and 45 °C. Irrelevant effects of temperature on BPA adsorption were reported by Xu et al. [42] in a temperature range of 25–45 °C. Conversely, Wang and Zhang [41] found a decrease of BPA adsorption when the temperature increased from 25 to 45 °C. 

**Figure 8:**
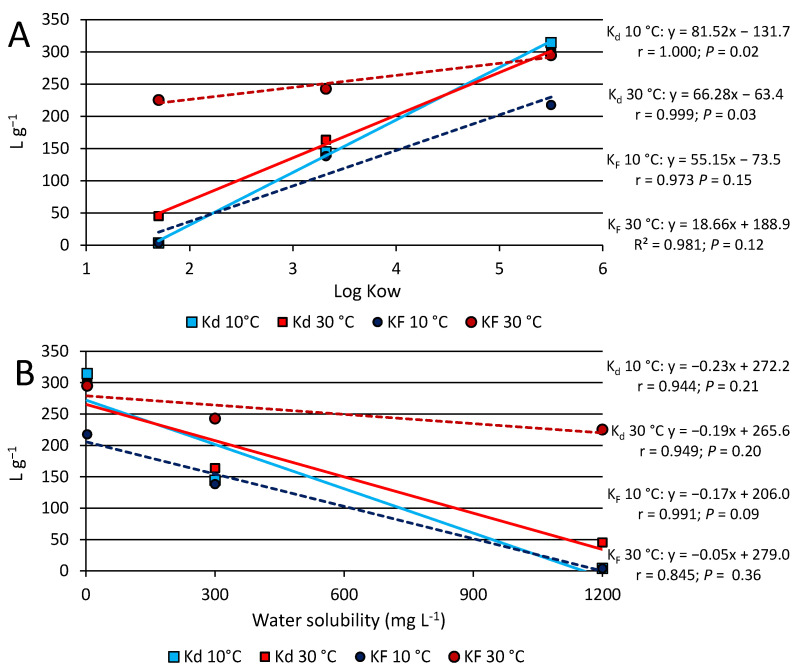
Plots of the correlations between the distribution coefficient, K_d_, and the Freundlich constant, K_F_, and the corresponding log Kow (**A**) and water solubility (**B**) of the compounds.

The level of correlation between the coefficients K_d_ and K_F_ and the corresponding Log Kow or water solubility of the compounds was explored through linear regression (Figure 8). At both temperatures tested, significant correlations (*p* ≤ 0.05) were found only between K_d_ and Log Kow values, while K_F_ values seemed less correlated with compound hydrophobicity (Figure 8A). Similarly, at both temperatures, only slight correlations were observed between K_d_ and K_F_ and water solubility (Figure 8B). These findings confirm the importance of hydrophobicity in the adsorption of organic chemicals on biochar.

**Figure 9:**
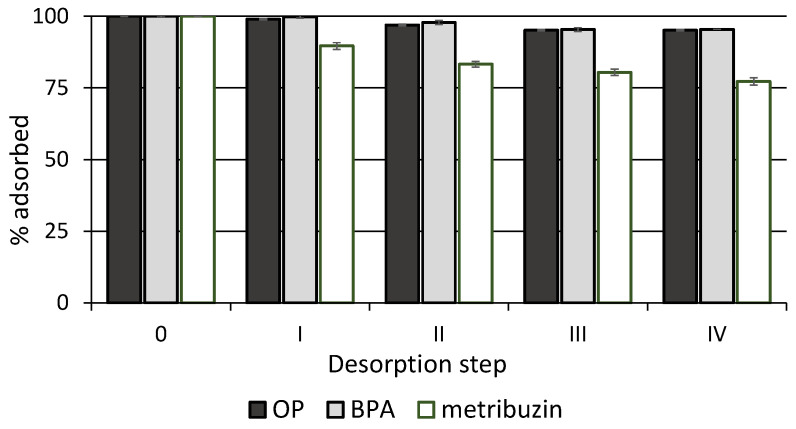
Percentage of compound that remains adsorbed on biochar after each desorption step. Standard error is reported as vertical bar on each point (n = 3).

Finally, desorption data of the compounds are shown in Figure 9. After four desorption steps, approximately 5, 5 and 23% of adsorbed OP, BPA and metribuzin were desorbed from biochar, respectively. Therefore, biochar showed an excellent ability to retain all compounds that were released very slowly and only negligibly (Figure 9). This was expected considering the physicochemical properties of the compounds and the high hydrophobicity of biochar. The occurrence of strong chemical interactions between biochar and the two phenolic EDCs could be the reason for the very low desorption rate and hysteresis phenomenon observed. The slightly higher desorption of metribuzin suggests a weaker interaction of this molecule with biochar, reasonably due to the formation of lower-energy bonds. Very little information is available in the literature on the desorption of these molecules from biochar, and most of the works concern regeneration studies. Choi and Kan [54] measured very low BPA desorption from an alfalfa biochar.

## 4. Conclusions

This study evaluated the potential of a biochar from poplar wood gasification to adsorb three EDCs, the xenoestrogens BPA and OP and the herbicide metribuzin. Biochar demonstrated excellent efficiency in removing all compounds, especially the more hydrophobic OP and BPA, from water. The quantity of compound removed was positively correlated with the solution/biochar ratio adopted. Adsorption kinetics study evidenced a very rapid sorption of all molecules, especially the two phenols that were retained almost instantaneously, according to a preferential pseudo-second order kinetic model. At both temperatures investigated (10 and 30 °C), the degree of adsorption followed the order: OP > BPA > metribuzin. The Freundlich model was the best at interpreting the equilibrium data and describing the sorption of each compound, although, in some cases, the Henry and the Temkin equations were also well suited. Compared to the results obtained in previous similar studies, the adsorption constants observed in the present work were one or two orders of magnitude higher, indicating an extraordinary retention efficacy of the biochar. The desorption rates of OP and BPA were very low, and much lower than that of metribuzin, denoting strong retention of the compounds on this material and the occurrence of hysteretic effects. A significant positive correlation was observed between the values of the distribution coefficient, K_d_, of the three compounds and the corresponding Log Kow values, thus confirming the prominent role of hydrophobicity in the sorption process. The overall results of this study encourage the use of biochar as a biosorbent of toxic organic chemicals in both agricultural and environmental contexts.

## Figures and Tables

**Table 1:** Some properties of biochar.

Parameter	Value
pH ^a^	9.97 ± 0.04 ^b^
Ash (%) ^c^	8.81 ± 0.16
EC (dS m^−1^) ^a^	3.83 ± 0.14
Elements (%)	
C	74.39 ± 0.50
H	0.86 ± 0.04
O	15.42 ± 0.45
N	0.52 ± 0.01
H/C (atomic ratio)	0.14 ± 0.01
O/C (atomic ratio)	0.16 ± 0.01
(O + N)/C (atomic ratio)	0.16 ± 0.01

Note: data are related to dry matter. Element content is on dry and ash-free basis. ^a^ 1:10 (*w*/*v*) in double distilled water; ^b^ values are the mean ± SD (n = 3); ^c^ at a temperature of 700 °C for 6 h.

**Table 2:** Theoretical models used.

Model	Equation	Parameters
**Adsorption Kinetics**		
PFO	q_t_ = q_e_ (1 − exp^−k1t^)	q_e_ and q_t_ (mg g^−1^) are the concentrations of the adsorbed compound at equilibrium and at time t, respectively, k_1_ (h^−1^) and k_2_ (g mg^−1^ h^−1^) are the rate constants of adsorption
PSO	$q_{t}=\frac{q_{e}^{2}k_{2}t}{1+{\;k}_{2}q_{e}t}$	
**Adsorption Isotherm**		
Freundlich	q_e_ = KF C_e_^1/n^	q_e_ (mg g^−1^) is the concentration of the adsorbed compound at equilibrium, C_e_ (mg L^−1^) is the equilibrium concentration of the compound in solution, 1/n indicates the degree of nonlinearity between the concentration of the compound in solution and that of the adsorbed compound, while the reciprocal n is the sorption intensity, K_F_ (L g^−1^) is the Freundlich adsorption constant, b (mg g^−1^) is the maximum adsorption capacity of the adsorbent, K_L_ (L g^−1^) is the Langmuir constant that expresses the energy of adsorption and the affinity of the solute for the adsorbent, Kd (L g^−1^) is the distribution coefficient
Langmuir	q_e_ = (K_L_Ce_b_) (1 + K_L_C_e_)	
Henry	q_e_ = Kd C_e_	
Temkin	q_e_ = B ln (A_T_ C_e_)	q_e_ (mg g^−1^) is the concentration of the adsorbed compound at equilibrium; C_e_ (mg L^−1^) is the equilibrium concentration of the compound in solution; A_T_ (L g^−1^) is the Temkin equilibrium binding constant, B (J mol^−1^) expresses the enthalpy of adsorption; B = RT/b_T_, where b_T_ is a constant related to the heat of adsorption; T is the absolute temperature (K) and R is the universal gas constant (8.314 J mol^−1^ K^−1^)

**Table 3:** Amount of compound adsorbed (mg g^−1^) as a function of the solution/biochar ratio adopted. One-way analysis of variance (ANOVA) and Duncan’s new multiple range test at *p* ≤ 0.01 (n = 3) were used for statistical treatment of data.

Compound	Ratio				
	500	1000	2000	5000	10,000
OP	0.99 ± 0.002 E	2.00 ± 0.001 D	3.73 ± 0.021 C	9.35 ± 0.013 B	19.12 ± 0.006 A
BPA	0.99 ± 0.005 D	1.84 ± 0.024 D	3.80 ± 0.057 C	8.88 ± 0.629 B	19.18 ± 0.030 A
Metribuzin	0.79 ± 0.021 E	1.84 ± 0.001 D	3.66 ± 0.007 C	8.81 ± 0.078 B	17.81 ± 0.047 A

Note: different letters indicate statistically significant differences at *p* ≤ 0.01.

**Table 4:** Kinetic pseudo-first order and pseudo-second order parameters obtained through the non-linear regression for the adsorption of the compounds onto the biochar.

Compound	Pseudo-First Order					Pseudo-Second Order			
	q_e, exp_ (mg g^−1^)	r	SSR	q_e,1_ (mg g^−1^)	k_1_ (h^−1^)	r	SSR	q_e2_ (mg g^−1^)	k_2_ (g mg^−1^ h^−1^)
OP	19.12	0.622	0.03	19.05	281.37	0.750	0.02	19.07	287.86
BPA	19.18	0.839	0.02	19.11	264.92	0.910	0.01	19.13	240.16
Metribuzin	17.51	0.758	9.35	16.64	82.52	0.895	4.59	17.18	8.34

**Table 5:** Adsorption parameters of the three compounds onto biochar.

		10 °C			30 °C		
Model		OP	BPA	Metribuzin	OP	BPA	Metribuzin
Henry	r	0.987	0.975	0.973	0.999	0.998	0.959
	SSR	12.9	22.2	2.1	0.7	2.3	29.5
	K_d_ (L g^−1^)	314.3	144.6	3.7	298.1	163.7	45.2
	K_OC_ (L g^−1^)	422.5	194.5	4.9	589.04	220.1	60.8
Freundlich	r	0.976	0.991	0.993	0.998	0.996	0.991
	SSR	10.4	4.5	0.1	0.7	0.6	2.8
	K_F_ (L g^−1^)	217.7	138.2	3.9	295.0	242.7	225.3
	1/n	0.88	1.86	0.58	1.00	1.16	2.17
Langmuir	r	0.943	0.974	0.941	0.996	0.994	0.833
	SSR	7.0	22.4	2.1	1.8	2.3	29.6
	b (mg g^−1^)	6562.4	3372.0	2.3	1149.9	4106.4	252.2
	K_L_ (L g^−1^)	0.06	0.02	1.11	2.30	0.04	0.01
Temkin	r	0.988	0.804	0.971	0.973	0.893	0.946
	SSR	5.7	95.7	0.5	28.5	43.2	44.6
	AT (L g^−1^)	178.7	230.1	16.2	246.6	140.7	16.4
	B (J mol^−1^)	7.60	3.32	1.45	5.77	5.33	7.99
	b_T_	309.5	696.3	1618.1	436.4	472.5	315.1

## Data Availability

Not applicable.
